# A Proteomics-Based Approach Reveals Differential Regulation of Urine Proteins between Metabolically Healthy and Unhealthy Obese Patients

**DOI:** 10.3390/ijms20194905

**Published:** 2019-10-03

**Authors:** Hicham Benabdelkamel, Afshan Masood, Meshail Okla, Mohammed Y. Al-Naami, Assim A. Alfadda

**Affiliations:** 1 Proteomics Resource Unit Obesity, Research Center, College of Medicine, King Saud University, Riyadh 11461, Saudi Arabia; hbenabdelkamel@ksu.edu.sa (H.B.); afsmasood@ksu.edu.sa (A.M.); 2 Department of Community Health Sciences, College of Applied Medical Sciences, King Saud University, 183T11, Riyadh 11495, Saudi Arabia; meokla@ksu.edu.sa; 3 Department of Surgery, College of Medicine, King Saud University, Riyadh 11472, Saudi Arabia; alnaami@ksu.edu.sa; 4 Department of Medicine, College of Medicine, King Saud University, Riyadh 11461, Saudi Arabia

**Keywords:** obesity, metabolic syndrome, healthy obese, unhealthy obese, urine proteins, proteomics

## Abstract

Metabolic dysfunction associated with obesity threatens to inundate health care resources by increasing the incidences of obesity-related diseases. The aim of the present study was to investigate the changes in the urinary proteome of 18 individuals classified into metabolically healthy obese (MHO) and metabolically unhealthy obese (MUHO) patients. Proteome analysis was performed using the two-dimensional difference in gel electrophoresis (2D-DIGE) coupled with mass spectrometry (MS). Upon analysis, a total of 54 proteins were found to be affected with ≥1.5-fold change (ANOVA, *p* ≤ 0.05), of which 44 proteins were upregulated and 10 proteins were downregulated. These differentially abundant proteins were related to nuclear factor κB (NF-κB) and p38 mitogen-activated protein (MAP) kinase pathways and were involved in cellular compromise, inflammatory response, and cancer. Proteins involved in inflammation (fibrinogen alpha (FIBA), serotransferrin (TRFE, and kininogen-1 (KNG1)) and insulin resistance (ADP-ribosylation factor (ARF)-like protein 15 (ARL15) and retinol-binding protein 4 (RET4)) were found to be significantly increased in the urine samples of MUHO compared to MHO patients. Investigating the effects of obesity on urinary proteins can help in developing efficient diagnostic procedures for early detection and prevention of obesity-related complications.

## 1. Introduction

Over the last 44 years, the worldwide prevalence of obesity has nearly tripled [1]. People who are obese, compared to those with a normal or healthy weight, are at increased risk of metabolic disorders such as insulin resistance, dyslipidemia, and hypertension. Obesity is also associated with increased risk of developing comorbidities including diabetes and cardiovascular diseases. However, accumulating evidence suggests that metabolically healthy obese (MHO) people were not at high risk of cardiovascular diseases as they displayed no metabolic abnormalities such as dyslipidemia, insulin resistance, hypertension, or a high degree of inflammation regardless of their high BMI [2,3]. An earlier study showed that a substantial proportion of overweight (≈50%) and obese (≈30%) individuals exhibited no metabolic and cardiovascular complications. MHO individuals demonstrated three clinical features—a reduced accumulation of visceral and ectopic fat for equal total adiposity, preserved insulin sensitivity, and a lower degree of systemic and adipose tissue inflammation compared to metabolically unhealthy obese (MUHO) people [2]. Thus, metabolically healthy and unhealthy obese individuals displayed different phenotypes [2,3].

Studying human obesity through the use of human samples from adipose tissue, blood, or other biological fluids is pertinent to understanding the molecular mechanisms and metabolic pathways leading to obesity or its associated comorbidities. For investigating renal diseases and other broad spectra of diseases, urine has been used as a diagnostic tool as it contains all the secreted proteins from tubules and the kidney as the result of blood filtration. For discovering new biomarkers or therapeutic targets, urinary proteomics has evolved as a promising platform for the identification of excreted proteins and peptides in different health conditions. Urine contains approximately 2000 proteins and has a less complex composition compared to plasma, which contains more than 10,000 core proteins. In addition, urine is considered as a safe and reliable alternative for biomarker studies compared to plasma and tissue samples because of the non-invasive collection procedure, the abundance of specimen, and the relatively stable content of peptides and proteins as it undergoes the complete proteolytic degradation process by endogenous proteases during its storage in the bladder [4].

Several reports on expression proteomics have shown that differential protein expression profiles exist in relation to different metabolic states [5,6,7]. In one study, a proteomic analysis of visceral fat tissue from obese people with and without metabolic syndrome (MetS) revealed that individuals characterized as MHO had an increased abundance of proteins related to adipose tissue expansion and antioxidant capacity. In comparison, the MUHO group showed increased abundance of proteins that related to mitochondrial and the lipid droplet metabolic activities [6]. In another study, 20 proteins were reported to be differentially expressed between MHO and MUHO individuals and most of these proteins were related to inflammatory and lipid processes [5]. Moreover, the analysis of circulating adipokines showed that MHO individuals had higher adiponectin levels compared to MUHO individuals [7]. These studies indicated that metabolic profiles varied in different health conditions and this affected the respective protein profiles. Thus, studying the different protein profiles between various obesity phenotypes for the discovery of novel biomarkers in obesity assessment can help in developing new therapeutic targets preventing the shift of individuals with MHO to MUHO. In the present study, we analyzed the urine proteome of MHO and MUHO patients using two-dimensional difference in gel electrophoresis (2D-DIGE) coupled with mass spectrometry (MS) [8,9]. Discriminating between MHO and MUHO individuals can aid the development of new pharmacological or dietary targets for alleviating health complications associated with obesity [2]. Furthermore, distinguishing between different obesity phenotypes may help in improving the current obesity treatment guidelines to enhance responsiveness of obese patients to medical interventions [3].

## 2. Results

### 2.1. Anthropometric and Biochemical Data

Two groups (MUHO and MHO) with similar BMI (BMI > 30 kg/m^2^) and age were chosen for this study. No significant differences were noted between the two groups with respect to body fat percentage, blood pressure, fasting glucose levels, lipid content, liver enzymes, and serum creatinine levels. The MUHO group was found to have higher insulin and homeostatic model assessment of insulin resistance (HOMA-IR) levels as compared to the MHO group (Table 1).

### 2.2. 2D-DIGE Analysis and Identification of Differentially Expressed Proteins

**Figure 1:**
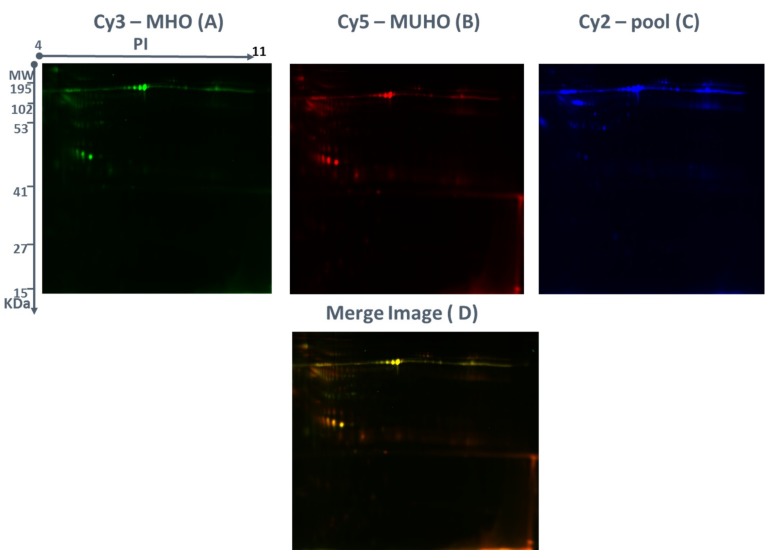
Representative fluorescent protein profiles using two-dimensional difference in gel electrophoresis (2D-DIGE) containing (**A**) a metabolically unhealthy obese (MUHO) sample labeled with Cy5, (**B**) a metabolically healthy obese (MHO) sample labeled with Cy3, and (**C**) a pooled internal control labeled with Cy2. Proteins were separated on IPG strip (pH 3–11) in the first dimension followed by 12.5% PAGE in the second dimension electrophoresis. Images were captured using a Typhoon 9400 Variable Mode. (**D**) An overlay of Cy3/Cy5 images is shown.

The MUHO and MHO groups’ protein expression profiles were studied by 2D-DIGE experiments with 9 biological replicates and yielded reproducible spot patterns for all the samples. Approximately, 1200 protein spots were mapped and the spots for the MUHO samples (red) and those of the MHO samples (green) were compared (Figure 1A–C). Urine protein levels were compared by 2D-DIGE between the MUHO and MHO urine samples. The gel images showed a high degree of differentially expressed proteins represented as yellow spots between the MHO and MUHO groups (Figure 1D). Yellow spots represent proteins with the same isoelectric point, molecular weight, and nearly equal fluorescence intensity.

**Figure 2:**
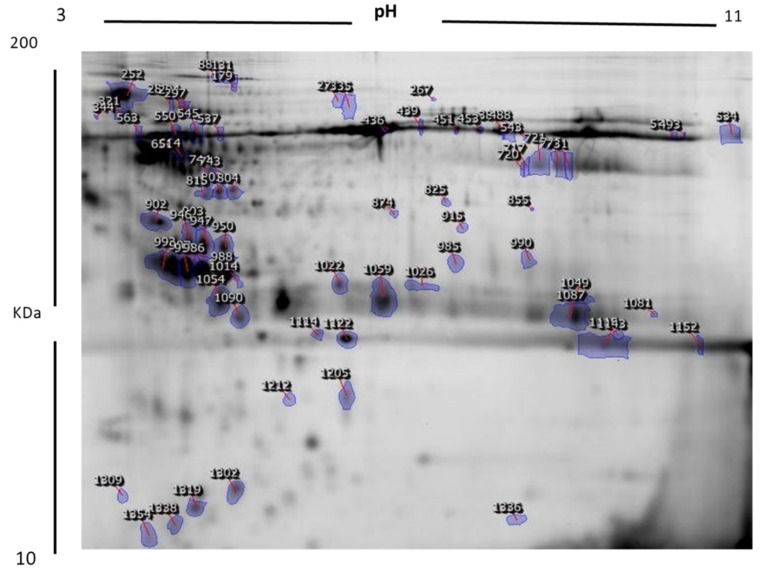
Representative image of protein spots from urine samples. Numbered spots indicate those which were identified with MALDI-TOF/TOF and were differentially expressed (over 1.5-fold change, *p* < 0.05).

Figure 2 shows a representative 2D-DIGE gel image from the set of pairwise comparisons that were made between the two groups. The arrow indicates the differentially abundant spots that are either decreased or increased (blue arrow) between the groups. The high reproducibility of the spot patterns across all the 9 gels permitted further analysis. Cy2 labeling (internal standard) was used for normalization across all the gels and for quantitative differential analysis of the protein levels. Significant changes in protein levels were analyzed by the ANOVA test (i.e., *p* ≤ 0.05 and fold change ≥1.5).

Progenesis statistical software analysis detected a total of 72 protein spots which showed a significant increase or decrease in the expression levels between the MHO and MUHO samples. In some cases, variants of the same protein were found at several locations on the gel. Peptide mass fingerprints (PMFs) identified 54 out of the 72 protein spots. Further, MALDI-TOF mass spectrometry found these 54 spots to be unique protein sequences and were matched to entries in the SWISS-PROT database by Mascot with high confidence scores. Out of 54 protein spots, 44 were increased and 10 were decreased in the MUHO group compared to the MHO group. We found several isoforms of the same proteins (KNG1, AMBP, albumin) and these findings were very typical of 2D-DIGE experiments, where the proteins may be isoforms or post-translational modifications that can shift the protein depending on the isoelectric point (pI) or the molecular weight (MW). Table 2 lists all 54 protein spots that were identified.

### 2.3. Mapping of Protein–Protein Interaction Networks

**Figure 3:**
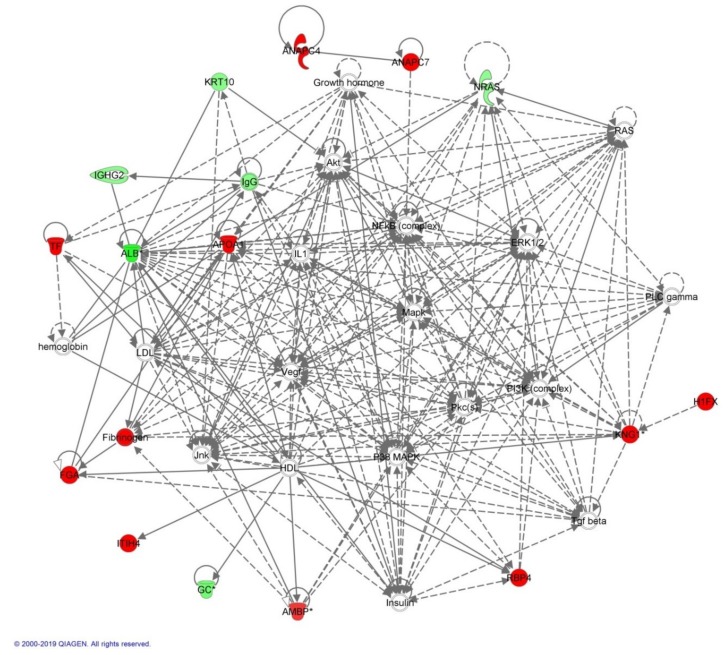
Schematic representation of the most significant Ingenuity Pathway Analysis (IPA) network of differentially regulated proteins in the MHO group compared to those from the MUHO group. IPA analysis indicated a functional interaction of networks, and the highest score of 40 was mostly related to cellular compromise, inflammatory response, and cancer. NF-κB and p38 mitogen-activated protein (MAP) kinases are indicated as central nodes that were deregulated in MUHO. Nodes in green and red correspond to downregulated and upregulated proteins in MHO vs. MUHO groups, respectively. Uncolored nodes are proposed by IPA and indicate potential targets that were functionally coordinated with the differentially expressed proteins. Solid lines indicate direct molecular interactions, and dashed lines represent indirect interactions.

The biological roles of the differentially abundant proteins identified in the MUHO group compared to the MHO group were investigated using Ingenuity Pathway Analysis (IPA) software. The software computes a score based on the best fit obtained from the input data set of proteins and from the biological functions database, to generate a protein–protein interaction network. The generated network is preferentially enriched for proteins with specific and extensive interactions; the interacting proteins were represented as nodes and their biological relationships as a line. Based on the data, two interaction networks were identified for the proteins exhibiting differential expression profiles. The highest scoring network (score = 40) (Figure 3) incorporated 17 focus molecules and were related to cellular compromise, inflammatory response, and cancer. 

The proposed highest interaction network pathway was related to cellular compromise, inflammatory response, and cancer. The three most interesting canonical pathways included liver X receptor (*p* = 6.21 × 10^−15^, 7.4% overlap), farnesoid X receptor (*p* = 6.9 × 10^−4^, 3.2 % overlap), and acute phase response (*p* = 3.35 × 10^−4^, 3.9% overlap). Details of the canonical pathways identified in this study are summarized in Figure S1 (Supplementary Materials).

### 2.4. Principal Component Analysis (PCA)

**Figure 4:**
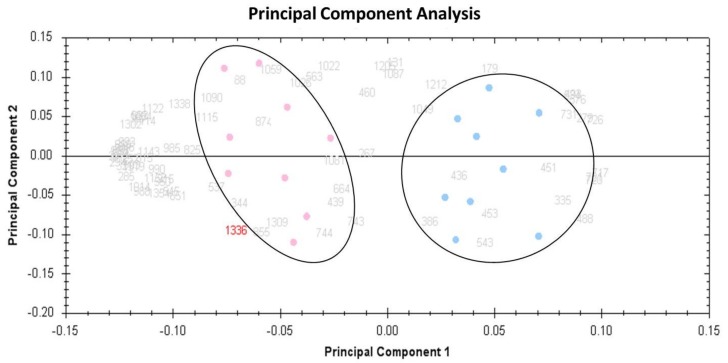
Principal component analysis (PCA) plot showing the two first principal components. Taken together, these explained 62 % of the selected spot’s variability values. Colored dots and numbers are the representation of gels and spots, respectively.

Multivariate analyses of the protein abundance data were performed using Progenesis Same Spots software (V 3.3, Nonlinear Dynamics, London, UK). The gel images were divided into two groups that included 9 gels from MUHO and 9 from MHO. The data were filtered to include only 72 spot features that exhibited statistically significant (ANOVA *p* < 0.05) changes in abundance, identified by MS. The analyses revealed that the two groups clustered distinctly from one another based on different urine proteins (Figure 4). 

### 2.5. Classification of Key Proteins Based on Their Function

**Figure 5:**
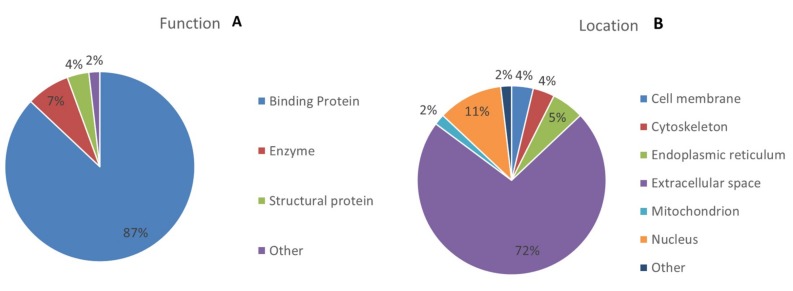
Comparative depiction (%) of identified proteins categorized into groups according to their function (**A**), and cellular component (**B**).

To gain a better understanding of the molecular function and biological implications of the 54 differentially abundant proteins identified between the MUHO and MHO groups, functional annotations were carried out based on Gene Ontology (GO) terms using UniProtKB (https://www.uniprot.org/). The dominant functional categories identified were for binding protein (87%), enzyme (7%), structural protein (4%), and others (3%). Figure 5 depicts the percent involvement of each of these proteins within their biological functional categories.

## 3. Discussion

In the present study, we compared the urinary proteomic profiles between obese people with or without metabolic disorders using the 2D-DIGE MALDI-TOF proteomics approach. Proteomic analysis of urinary proteins may contribute towards a better understanding of the differences in the overall metabolism observed between healthy and unhealthy obese people. Overall, our results showed that a total of 54 proteins were affected in the metabolic state associated with obesity with a ≥1.5-fold change, of which 44 proteins were upregulated and 10 proteins were downregulated. The differentially abundant proteins were mostly related to NF-κB and p38 mitogen-activated protein (MAP) kinase pathways and were involved in cellular compromise, inflammatory response, and cancer. Our data showed that there was a significant increase in the levels of proteins involved in inflammation FIBA, TRFE, and KNG1 and insulin resistance ARL15 and RET4 in the urine samples of the MUHO group compared to the MHO group.

### 3.1. Fibrinogen Alpha Chain (FIBA)

Fibrinogen plays a role in fibrin formation, which is one of the primary components of blood clots. Fibrinogen stimulates chemokine secretions via Toll-like receptor 4 and thus facilitates mounting of the immune response in mouse macrophage-like (RAW264.7) and human monocytic (U937 and THP-1) cell lines [10]. We found that urine samples of the MUHO group showed a 1.7-fold increase in FIBA compared to the MHO group. These findings were consistent with the observation that the protein expression profile of plasma between non-obese and obese individuals exhibited an overexpression of fibrinogen in the obese group [11]. Obesity elicited a chronic, low-grade systemic inflammatory response that was due to a combination of increased insulin resistance and an increased production of inflammatory mediators by expanding the pool of adipocytes [12]. Fibrinogen was recognized as one of the inflammation-sensitive plasma proteins (ISPs) that positively correlated with body fat mass and inflammatory markers [11,13]. It has been proposed that proinflammatory cytokines formed in the adipose tissue may increase the hepatic synthesis of ISPs [9]. Therefore, the elevated levels of FIBA in urine samples of MUHO compared to MHO mostly reflect the existence of inflammatory conditions and the excess production of ISPs.

### 3.2. Serotransferrin (TRFE)

Transferrins transport iron from sites of absorption and heme degradation to those of storage and utilization [14,15]. In our study, the MUHO group showed a 1.5-fold increase in urinary excreted TRFE compared to the MHO group. The low-grade chronic inflammatory response associated with obesity may contribute to the TRFE changes observed in the MUHO group. TRFE acts as a negative acute phase reactant whose levels are reduced during inflammation to limit the circulating iron and to prompt the sequestration of iron in macrophages to modulate host defenses [15,16]. We think that a positive relationship exists between the severity of obesity and urinary TRFE, because we previously noted a significant reduction in the urinary TRFE levels in obese people in response to either a low-calorie diet or bariatric surgery [17]. Therefore, the elevated urinary TRFE levels observed in the MUHO group may be due to the rapid filtration of TRFE to maintain low serum levels in the inflammation state associated with obesity. Alternatively, transferrinuria may indicate the presence of glomerular damage in MUHO individuals as it is a sensitive marker for early proteinuria and increased vascular permeability [14,18].

### 3.3. Kininogen-1 (KNG1)

The human genome contains a single copy of the kininogen gene, which is responsible for the production of kininogens, both high-molecular-weight (HK) and low-molecular-weight (LK) [19]. HK plays an essential role in the assembly of the plasma kallikrein–kinin system (KKS), which contains two serine proteases and an HK. Upon stimulation of the KKS, HK is cleaved to release bradykinin, a potent inflammatory mediator that causes vasodilation and enhanced capillary permeability. In addition, bradykinin may mediate the angiogenesis effect of HK [19,20]. The KKS system is also activated in response to many physiological and pathophysiological conditions [20], including blood coagulation, regulation of blood pressure, pain, and inflammation and may contribute to arterial thrombosis, sepsis, and Alzheimer’s disease [19]. KNG1 played a critical role in the pathogenesis of colitis in an animal mouse model for inflammatory bowel disease. In these mice, lack of KNG1 reduced colitis development, infiltration of inflammatory cells into the gut mucosa, and inflammatory cytokine levels (e.g., TNFα, IFN-γ, IL-1β, and IL-6) [20]. Recently, the role of KNG1 in energy metabolism was discovered. In rodents, KNG1 was suggested to have a thermogenic function in the subcutaneous adipose tissue where it was induced by post-cold stimulation. In addition, KNG1 was induced in mice with impaired adaptive thermogenesis in the brown adipose tissue [21,22]. We found a 1.6-fold increase in the urinary excretion of KNG1 in MUHO group compared to MHO group. This may possibly reflect the elevated levels of kininogen production due to the immune system activation in MUHO people. Alternatively, high levels of KNG1 in the MUHO group may be the result of an adaptive response to counter the impaired metabolism in adipose tissue in individuals with severe obesity.

### 3.4. ADP-Ribosylation Factor (ARF)-Like Protein 15 (ARL15)

ARL15 is a member of the ARF-family of proteins involved in the regulation of vesicle trafficking and biogenesis [23]. In addition, ARL15 was identified recently as a new candidate gene related to diabetes [24]. The gene was highly expressed in β-cells and had a potential role in the regulation of β-cells and insulin secretion. ARL15 played a role in type 2 diabetes susceptibility and fasting insulin regulation [25,26]. In addition to the direct relationship between ARL15 and diabetes, an association between ARL15 and diabetes-related atherosclerosis has been reported [24]. Although the relationship between ARL15 and diabetes has been reported, the mechanism of how ARL15 is associated with insulin resistance remains unknown. One study showed that ARL15 acted as an insulin-sensitizing effector molecule to activate the phosphorylation of members of the canonical insulin signaling pathway. Although ARL15 was upregulated by insulin stimulation, this effect was impaired in insulin-resistant muscles [24]. Therefore, ARL15 may either play a causative role in insulin resistance or an adaptive response to enhance insulin action. In our study, we found that MUHO patients excreted 2.1-fold higher amounts of ARL15 than MHO patients in the urine. The increase in ARL15 levels in the urine of MUHO patients may have resulted from the elevated levels of insulin secreted to compensate for reduced insulin sensitivity [24]. In addition, higher levels of ARL15 in urine may help in the prediction of the increased risk of diabetes in MUHO individuals.

### 3.5. Retinol-Binding Protein 4 (RET4)

RET4 is a specific transport protein for retinol which delivers retinol from the liver stores to the peripheral tissues. RET4 was identified as an adipokine that linked obesity to insulin resistance [27]. In adipose-Glut4 knockout mice, RET4 was induced in adipose tissue. Moreover, serum RET4 was induced with insulin resistance in mice and humans. The negative relationship between RET4 and insulin sensitivity was further verified in mice that developed insulin resistance upon overexpression of RET4, whereas insulin action was improved upon RET4 depletion. In addition, the insulin-sensitizing antidiabetic agent, rosiglitazone, normalized RET4 levels. Although the mechanism of how RET4 affects insulin sensitivity is not clear, RET4 may cause insulin resistance by inducing the gluconeogenic enzyme, phosphoenolpyruvate carboxykinase, in the liver and by impairing insulin signaling in muscles [28]. In our study, we found that RET4 was affected by the metabolic state associated with obesity, as the MUHO group showed 1.5-fold higher levels of excreted RET4 in urine compared to the MHO group. The elevated levels of RET4 indicated an increase in serum RET4 in MUHO compared to MHO and it reflected the altered insulin sensitivity in MUHO people.

## 4. Materials and Methods

### 4.1. Ethical Approval and Consent to Participate

Prior to implementation, all procedures and protocols were reviewed and approved by the Institutional Review Board, College of Medicine, King Saud University No. 08/1960/E. Written informed consent to use the samples for research was obtained from all the participants. This study was conducted at the Obesity Research Center, College of Medicine, King Saud University, Riyadh, Saudi Arabia.

### 4.2. Study Design and Patient Selection

A group of 18 obese patients (9 women and 9 men) aged between 24 and 40 years were selected from a cohort of Saudi patients undergoing bariatric surgery at the King Khalid University Hospital, King Saud University, Riyadh. Weight (in kilograms) and height were measured using a stadiometer and BMI was calculated. The BMIs ranged from 34.9 to 61.9 kg/m^2^. A body fat analyzer (Gima Spa, Milan, Italy) was used to calculate the fat percentage and lean mass. The patients were subdivided into two groups based on the presence or absence of comorbidities as defined by the International Diabetes Federation (IDF). The components of IDF include presence of central obesity (or BMI > 30 kg/m^2^) along with two or more of the following criteria: fasting triglycerides level ≥1.7 mmol/L; high density lipoprotein (HDL) cholesterol level <1.04 mmol/L in men or <1.3 mmol/L in women or administration of lipid-lowering medication; systolic/diastolic blood pressure ≥130/85 mmHg or administration of antihypertensive medication; and fasting glucose level ≥5.56 mmol/L or administration of antidiabetic medication. Accordingly, 9 patients were considered to be MUHO when they had at least three components of MetS or more including central obesity, whereas 9 patients were classified as MHO if they had a single component of MetS or less including central obesity (see Table S1, Supplementary Materials). All the patients were on lifestyle modification and did not receive any treatment. The exclusion criteria included the presence of acute inflammation, infection, or malignancy.

### 4.3. Blood Collection and Biochemical Assays

Blood samples were taken under fasting conditions at the time of the operation. Plasma was separated immediately by centrifugation and aliquots were frozen at −80 °C for subsequent analysis. Biochemical investigations were carried out on all the patients′ samples prior to the operation. The levels of serum glucose, triglycerides, total cholesterol, and HDL cholesterol were determined using a Dimension Xpand Plus integrated clinical chemistry autoanalyzer (Siemens Healthcare Diagnostics, Deerfield, IL, USA). The serum levels of low-density lipoprotein (LDL) cholesterol were calculated using Friedewald’s equation. The plasma insulin concentration was determined by electro-chemiluminescence on a Cobas e411 immunoanalyzer (Roche Diagnostics, Indianapolis, IN, USA). Insulin resistance was determined using the HOMA-IR, which was calculated according to the following equation: HOMA-IR = fasting plasma glucose (mmol/L) × fasting plasma insulin (mIU/L) ÷ 22.5. The percentage of glycosylated hemoglobin (HbA1c) was measured using a turbidimetric inhibition immunoassay on a Dimension Xpand Plus autoanalyzer (Siemens Healthcare Diagnostics, Deerfield, IL, USA).

### 4.4. Urine Collection and Protein Extraction

For every patient, a midstream spot urine sample (50–100 mL) was collected in a clean catch specimen into a sterile urine container and immediately transported on ice to prevent microbe contamination and proteolysis. The samples were then processed and insoluble materials were removed by centrifugation at 2000× *g* (4000 rpm) at 4 °C for 10 min, within 30 min of collection, to prevent protein release from these artifacts. The supernatants were carefully removed and frozen at −80 °C in 2 mL aliquots for long-term storage. Proteins were isolated from the urine samples by precipitation with methanol and chloroform (i.e., 4 parts of methanol and 1 part of chloroform to 1 part sample) as previously described [29]. The protein pellets were solubilized in labeling buffer (7 M urea, 2 M thiourea, 30 mM Tris–HCl, and 4% CHAPS, pH 8.5). Insoluble material was pelleted by centrifugation (12,000 g, room temperature, 5 min), and protein concentrations were determined in triplicate using the 2D-Quant kit (GE Healthcare, Piscataway, NJ, USA), and the pH of the samples was adjusted to 8.5 using NaOH (100 mM).

### 4.5. Protein Labeling with Cyanine Dyes

The proteins were labeled with 400 pmol of CyDye™ DIGE Fluor dyes (GE Healthcare, Buckinghamshire, UK) in 1 μL of DMF and mixed with 50 μg of protein. The mixtures were incubated on ice for 30 min in a dark place. Afterward, the labeling reaction was terminated by adding 1 μL of 10 mM lysine. Each technical duplicate of the sample was covalently labeled with a fluorophore, either Cy3 or Cy5. A mixture of equal amounts of protein isolated from each sample in the experiment was labeled with Cy2 and used as an internal standard (Table S2, Supplementary Materials).

### 4.6. Two-Dimensional Electrophoresis, Image Scanning, and Preparative Gel

First dimension analytical gel electrophoresis was performed as follows. Nine immobiline Dry Strips (24 cm, pH 3-11; GE Healthcare, Uppsala, Sweden) were passively rehydrated (30 V, 12 h). This was followed by isoelectric focusing using an Ettan IPGphor IEF unit (GE Healthcare, Uppsala, Sweden). Focusing was performed at 20 °C and 50 μA per strip, according to the following steps and hold sequence: 500 V for 1 h, 1000 V for 1 h, 8000 V for 3 h, 8000 V up to a total of 45,000 V for 22 h. After the first dimension, the strips were equilibrated and separated on 12.5 % (SDS-PAGE) gels using an Ettan Dalt Six device (GE Healthcare, Uppsala, Sweden). The gels were scanned with a Typhoon 9400 scanner (GE Healthcare, Uppsala, Sweden) using appropriate wavelengths and filters for Cy2, Cy3, and Cy5 dyes. Total protein (1 mg) was obtained from a pool of equal protein amounts of each sample. This was denatured in lysis buffer, then mixed in a rehydration buffer. Gels were then stained by Colloidal Coomassie Blue staining.

### 4.7. Protein Identification by MALDI-TOF Mass Spectrometry (MS)

The spots from Coomassie-stained gels were excised manually, washed, and digested according to a previously published protocol [30]. The mixture of tryptic peptides (1 µL), derived from each protein, was spotted onto a MALDI target (384 MTP 800 µm AnchorChip; Bruker Daltonik, Bremen, Germany), together with 0.8 μL of matrix (10 mg α-cyano-4-hydroxycinnamic acid (CHCA) in 1 μL of 30% CH3CN and 0.1% TFA, and then left to dry at room temperature before mass spectrometry (MS) analysis. Spectra were acquired using a MALDI-TOF MS (UltraFlexTrem, Bruker Daltonik, Bremen, Germany) in the positive mode with a target voltage of 25 kV and pulsed ion extraction voltage of 20 kV. The reflector voltage was set to 21 kV and a detector voltage to 17 kV. PMF was calibrated against a standard (Peptide Calibration Standard II, Bruker Daltonik, Bremen, Germany). The PMF was processed using the Flex AnalysisTM software (version 2.4, Bruker Daltonik, Bremen, Germany). The MS data were interpreted using BioTools v3.2 (Bruker Daltonik, Bremen, Germany), together with the Mascot search algorithm (version 2.0.04 updated 09/05/2017; Matrix Science Ltd., city, UK). Mascot search parameters were set as follows: fixed propionamide modification of cysteine, oxidation of methionine as variable modification, one missed cleavage site (such as in the case of incomplete trypsin hydrolysis), and a mass tolerance of 100 ppm. Identified proteins were accepted if they showed a Mascot score greater than 56 and *p* < 0.05. Not all spots of interest could be identified because some proteins were low in abundance and did not yield a sufficiently intense mass of fingerprints; other spots were mixtures of multiple proteins.

### 4.8. Image Acquisition and Statistical Analysis

DIGE images were analyzed using Progenesis Same Spots v3.3 software (Nonlinear Dynamics Ltd., London, UK). First, images were aligned. Prominent spots were used to manually assign 40 vectors to digitized images within each gel and then the automatic vector tool was used to add additional vectors (350 total vectors), which were manually revised and edited for correction if necessary. These vectors were used to warp and align gel images with a reference image of one internal standard across and within each gel. Gel groups were established according to the experimental design and spot normalized volume was used to select statistically significant spots. The software calculated the normalized volume of each spot on each gel from Cy3 (or Cy5) to the Cy2 spot volume ratio. The software performed a log transformation of the spot volumes to generate normally distributed data. Log normalized volume was used to quantify differential expression. Independent direct comparisons were made between MUHO versus MHO, and fold differences and *p*-values were calculated using one-way ANOVA. All spots were pre-filtered and manually checked before applying the statistical criteria (ANOVA test, *p* ≤ 0.05 and fold ≥ 1.5). Normalized spot volumes, instead of spot intensities, were used in statistical processing. Spots fulfilling the above-mentioned statistical criteria were submitted for MS analysis.

### 4.9. Pathway Analysis

Pathway analysis was carried out by importing the quantitative data into the Ingenuity Pathway Analysis (IPA) software (license of 2019, Ingenuity^®^ Systems, Redwood, CA, USA, http://www.ingenuity.com). This software helped in determining the functions and pathways that were most strongly associated with the proteins listed by overlaying the experimental expression data on networks constructed from published interactions.

### 4.10. Statistical Analyses

The results for the biochemical parameters in the MHO and MUHO groups were presented as mean ± SD, and significant differences between the mean values were assessed using Student′s *t*-test. All statistical analyses were conducted using Graph Pad Prism software, version 5.0 for Windows (GraphPad Software, San Diego CA, USA).

## 5. Conclusions

In conclusion, by employing two-dimensional gel electrophoresis followed by mass spectrometry, we performed urinary proteomics for metabolically healthy and unhealthy obese individuals. A total of 54 proteins showed different expression profiles. These differentially abundant proteins were related to NF-κB and p38 MAP kinase pathways and were involved in cellular compromise, inflammatory response, and cancer. Proteins involved in inflammation (FIBA, TRFE, and KNG1) and insulin resistance (ARL15 and RET4) were abundantly excreted in the urine samples of metabolically unhealthy obese individuals compared to the metabolically healthy obese individuals. Further validation is needed to establish these proteins as urinary biomarkers for monitoring the metabolic state of obese individuals. In addition, these proteins could be utilized to evaluate the response to nutritional, medical, and surgical interventions for treating obesity.

## Figures and Tables

**Table 1:** Clinical, biochemical, metabolic, and body composition characteristics of study participants.

	MHO	MUHO	*p*-Value
N (male%)	9 (55.6%)	9 (44.4%)	
Age (years)	31.3 ± 6.5	32 ± 8.8	0.86
Weight (kg)	128.2 ± 37.3	131.2 ± 26.5	0.85
BMI (kg/m^2^)	48.1 ± 13.18	48.66 ± 8.8	0.92
Fat percentage	52.2 ± 15.7	55.8 ± 11.3	0.61
SBP (mm/Hg)	122.6 ± 12.4	129 ± 5.7	0.20
DBP (mm/Hg)	74.3 ± 7.9	72.9 ± 4.9	0.67
Fasting glucose (mmol/L)	7.0 ± 2.4	9.6 ± 4.1	0.17
Insulin (mIU/mL)	15.0 ± 3.9	24.4 ± 10.9	0.05
HOMA-IR	4.6 ± 2.2	10.7 ± 7.5	0.05
Total cholesterol (mmol/L)	4.4 ± 1.0	4.4 ± 0.6	0.87
Triglycerides (mmol/L)	1.4 ± 0.8	2.0 ± 1.6	0.34
HDL cholesterol (mmol/L)	1.0 ± 0.3	0.8 ± 0.1	0.11
LDL cholesterol (mmol/L)	2.7 ± 0.8	2.7 ± 0.7	0.93
Aspartate transaminase (IU/L)	25.6 ± 13.9	25.8 ± 13.8	0.97
Alanine transaminase (IU/L)	41.0 ± 19.8	44.1 ± 28.0	0.80
Alkaline phosphatase (IU/L)	77.3 ± 24.0	92.9 ± 15.9	0.17
Creatinine (mmol/L)	72.5 ± 13.1	76.1 ± 23.3	0.71

**Table 2:** List of differentially expressed proteins identified in urine between MUHO vs. MHO groups using 2D-DIGE with differences in fold change. Protein name, accession number, Mascot score, MS % coverage, MW, and pI values are listed according to the UniProt database (https://www.uniprot.org/).

Spot No.	Accession No. ^a^	Mascot ID	Protein Name	pI ^b^	MW ^c^	Cov%	Score ^d^	*p*-Value	Fold Change ^e^	MUHO/MHO Ratio Up or Down
980	P01042	KNG1	Kininogen-1	6.34	72,996	32	122	0.03	1.6	Up
874	P02768	ALBU	Serum albumin	5.92	71,317	41	97	0.026	1.5	Up
451	P02768	ALBU	Serum albumin	5.92	71,317	41	94	0.042	3	Down
985	Q6ZN57	ZFP2	Zinc finger protein 2 homolog	8.91	54,360	14	56	0.0163	2.1	Up
563	P02768	ALBU	Serum albumin	5.92	71,317	40	86	0.02	1.5	Up
744	P02768	ALBU	Serum albumin	5.92	71,317	32	70	0.022	1.65	Down
664	P02774	VTDB	Vitamin D-binding protein	5.4	54,526	29	66	0.018	1.9	Down
1081	P02768	ALBU	Serum albumin	5.92	71,317	34	106	0.026	2.1	Up
1087	APOA1	APOA1	Apolipoprotein A-I	5.56	30,759	37	78	0.04	1.5	Up
1090	P49753	ACOT2	Acyl-coenzyme A thioesterase 2, mitochondrial	8.7	53,584	18	56	0.05	1.5	Up
1059	Q92522	HIX	Histone H1x	10.76	22,474	17	56	0.05	1.7	Up
1049	Q8IVL5	P3H2	Prolyl 3-hydroxylase 2	5.48	81,846	30	56	0.04	1.5	Up
1049	Q9Y680	FKBP7	Peptidyl-prolyl cis-trans isomerase FKBP7	6.09	30,332	28	56	0.031	1.6	Up
743	P02768	ALBU	Serum albumin	5.92	71,317	22	74	0.039	1.7	Down
1302	Q8N720	ZN655	Zinc finger protein 655	6.7	58,682	26	56	0.047	1.8	Up
903	Q14624	ITIH4	Inter-alpha-trypsin inhibitor heavy chain H4	6.51	103,521	11	60	0.03	1.5	Up
902	P02671	FIBA	Fibrinogen alpha chain	5.7	93,636	20	58	0.05	1.7	Up
947	P02760	AMBP	Protein AMBP	5.95	39,886	27	93	0.037	1.7	Up
946	P02760	AMBP	Protein AMBP	5.95	39,886	33	70	0.04	1.5	Up
950	P02760	AMBP	Protein AMBP	5.95	39,886	20	56	0.049	1.6	Up
1122	Q9NS00	C1GLT	Glycoprotein-N-acetylgalactosamine 3-beta-galactosyltransferase 1	6.17	42,632	29	58	0.0385	2.1	Up
803	P02768	ALBU	Serum albumin	5.92	71,317	24	59	0.05	1.5	Up
815	P02768	ALBU	Serum albumin	5.92	71,317	40	75	0.05	1.5	Up
453	P02768	ALBU	Serum albumin	5.92	71,317	43	115	0.039	1.8	Up
454	P02768	ALBU	Serum albumin	5.92	71,317	14	62	0.05	1.5	Up
460	P02768	ALBU	Serum albumin	5.92	71,317	32	132	0.047	1.6	Up
1143	P02768	ALBU	Serum albumin	5.92	71,317	40	94	0.043	1.6	Up
985	Q9NXU5	ARL15	ADP-ribosylation factor-like protein 15	5.41	23,261	31	56	0.016	2.1	Up
943	P02760	AMBP	Protein AMBP	5.95	39,886	24	60	0.045	1.8	Up
986	Q9UJX3	APC7	Anaphase-promoting complex subunit 7	5.5	63,720	23	58	0.05	1.6	Up
987	P02760	AMBP	Protein AMBP	5.95	39,886	24	60	0.05	1.5	Up
992	P02760	AMBP	Protein AMBP	5.95	39,886	26	56	0.004	32.1	Up
534	P02768	ALBU	Serum albumin	5.92	71,317	32	122	0.05	1.7	Up
1152	Q8TD57	DYH3	Dynein heavy chain 3, axonemal	6.04	473,776	8	65	0.05	1.6	Up
537	P02768	ALBU	Serum albumin	5.92	71,317	42	128	0.05	1.6	Down
1022	P02768	ALBU	Serum albumin	5.92	71,317	30	58	0.05	1.5	Up
131	P02768	ALBU	Serum albumin	5.92	71,317	52	132	0.05	1.5	Up
545	P02768	ALBU	Serum albumin	5.92	71,317	48	114	0.05	1.6	Up
1026	P02768	ALBU	Serum albumin	5.92	71,317	39	81	0.05	1.5	Up
550	P02768	ALBU	Serum albumin	5.92	71,317	31	130	0.05	1.5	Up
1114	Q3ZCX4	ZN568	Zinc finger protein 568	8.58	76,601	34	56	0.05	1.8	Up
1090	P02753	RET4	Retinol-binding protein 4	5.76	20,007	63	64	0.05	1.5	Up
721	Q9Y680	FKBP7	Peptidyl-prolyl cis-trans isomerase FKBP7	6.99	30,332	38	56	0.05	1.6	Down
294	P02768	ALBU	Serum albumin	5.92	71,317	45	109	0.05	1.5	Up
1054	Q9UJX5	APC4	Anaphase-promoting complex subunit 4	5.36	92,896	17	58	0.04	1.5	Up
321	Q96M60	COO33	Protein FAM227B	9	60,317	17	58	0.05	1.6	Up
651	P02774	VTDB	Vitamin D-binding protein	5.4	54,526	49	75	0.05	1.6	Down
1212	P01111	RASN	GTPase NRas	5.01	21,501	38	59	0.04	1.5	Down
720	P01859	IGHG2	Immunoglobulin heavy constant gamma 2	7.66	36,515	33	56	0.05	1.6	Down
285	P02787	TRFE	Serotransferrin	6.81	79,280	40	93	0.05	1.5	Up
726	P13645	K1C10	Keratin, type I cytoskeletal 10	5.13	59,020	25	52	0.05	1.6	Down
386	P02768	ALBU	Serum albumin	5.92	71,317	45	109	0.01	1.5	Up
974	P02768	ALBU	Serum albumin	5.92	71,317	45	109	0.03	2.5	Up
971	P01042	KNG1	Kininogen-1	6.34	72,996	30	66	0.017	2	Up

^a^—Protein accession number for SWISS-PROT Database. ^b^—Theoretical isoelectric point. ^c^—Theoretical relative mass. ^d^—Mascot score. ^e^—Protein expression between MHO and MUHO states.

## Supplementary Materials

Supplementary materials can be found at https://www.mdpi.com/1422-0067/20/19/4905/s1.
