# Supplementary material for: A Proteomics-Based Approach Reveals Differential Regulation of Urine Proteins between Metabolically Healthy and Unhealthy Obese Patients
Source: Int J Mol Sci. 2019 Oct 3;20(19):4905. doi: 10.3390/ijms20194905 (PMC6801506; doi:10.3390/ijms20194905)
Supplement: Supplementary file 1 [file ijms-20-04905-s001.pdf]

Supplementary Materials:

	BMI	GLUC >5.6 (mmol/L)	TGL (>1.7mmol/L)	HDL <1.03 mmol/L in males and <1.29 mmol/L in females)	BP mm/Hg Systolic >130 or Diastolic >85	
**MUHO**						
Female	34	Y	Y	N	N	N
Male	39	N	N	Y	Y	N
Female	56	Y	N	Y	N	N
Female	59	Y	N	Y	Y	N
Male	42	N	N	Y	Y	N
Male	57	Y	Y	Y	N	N
Female	42	Y	N	Y	Y	N
Male	52	Y	Y	Y	Y	N
Male	57	Y	Y	Y	N	N
**MHO**						
Female	68.2	Y	N	N	N	N
Male	71.9	Y	N	N	N	N
Female	37.6	Y	N	N	N	N
Male	50.0	N	Y	N	N	N
Female	37.8	N	Y	N	N	N
Female	40.2	N	N	Y	N	N
Female	32.9	N	Y	N	N	N
Male	40.9	Y	N	N	N	N
Male	53.4	N	N	Y	N	N

Y – means Yes, the patient has risk factor
N – means No , the patient does not have risk factor.

Table S1: Table showing the grouping of patients into MHO and MUHO according to the IDF criteria.

Gel	Cy3	Cy5	Cy2
1	MHO-1	MUHO-10	Pooled sample
2	MUHO-11	MHO-2	Pooled sample
3	MHO-3	MUHO-12	Pooled sample
4	MHO-4	MUHO-13	Pooled sample
5	MUHO-14	MHO-5	Pooled sample
6	MUHO-15	MHO-6	Pooled sample
7	MHO-7	MUHO-16	Pooled sample
8	MHO-8	MUHO-17	Pooled sample
9	MUHO-18	MHO-9	Pooled sample

Table S2: Outline for the experimental design.

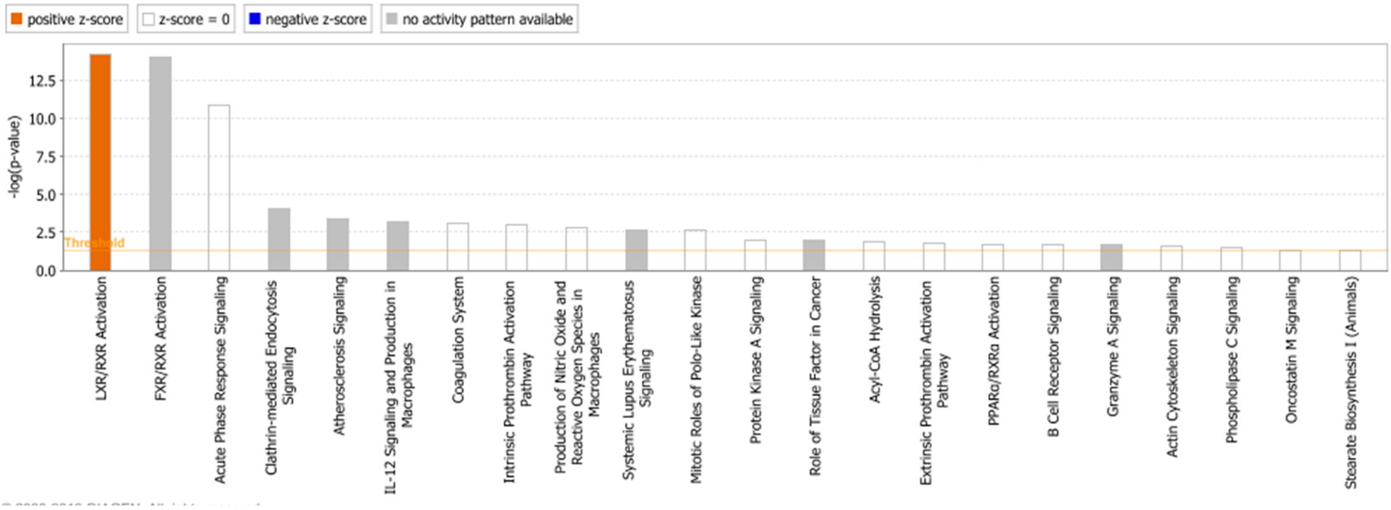

Figure S1: The figure shows the different canonical pathways obtained from IPA functional analysis.
